# Water deprivation induces hypoactivity in rats independently of oxytocin receptor signaling at the central amygdala

**DOI:** 10.3389/fendo.2023.1062211

**Published:** 2023-01-31

**Authors:** Viviane Felintro, Verónica Trujillo, Raoni C. dos-Santos, Claudio da Silva-Almeida, Luís C. Reis, Fábio F. Rocha, André S. Mecawi

**Affiliations:** ^1^ Department of Physiological Sciences, Instituto de Ciências Biológicas e da Saúde, Universidade Federal Rural do Rio de Janeiro, Seropédica, Brazil; ^2^ Department of Physiology, Facultad de Ciencias Exactas, Físicas y Naturales, Universidad Nacional de Córdoba, Córdoba, Argentina; ^3^ Department of Biophysics, Escola Paulista de Medicina, Universidade Federal de São Paulo, São Paulo, Brazil

**Keywords:** central amygdala, oxytocin receptor, dehydration, exploratory behavior, elevated plus maze test

## Abstract

**Introduction:**

Vasopressin (AVP) and oxytocin (OXT) are neuropeptides produced by magnocellular neurons (MCNs) of the hypothalamus and secreted through neurohypophysis to defend mammals against dehydration. It was recently demonstrated that MCNs also project to limbic structures, modulating several behavioral responses.

**Methods and Results:**

We found that 24 h of water deprivation (WD) or salt loading (SL) did not change exploration or anxiety-like behaviors in the elevated plus maze (EPM) test. However, rats deprived of water for 48 h showed reduced exploration of open field and the closed arms of EPM, indicating hypoactivity during night time. We evaluated mRNA expression of glutamate decarboxylase 1 (Gad1), vesicular glutamate transporter 2 (Slc17a6), AVP (Avpr1a) and OXT (Oxtr) receptors in the lateral habenula (LHb), basolateral (BLA) and central (CeA) amygdala after 48 h of WD or SL. WD, but not SL, increased Oxtr mRNA expression in the CeA. Bilateral pharmacological inhibition of OXTR function in the CeA with the OXTR antagonist L-371,257 was performed to evaluate its possible role in regulating the EPM exploration or water intake induced by WD. The blockade of OXTR in the CeA did not reverse the hypoactivity response in the EPM, nor did it change water intake induced in 48-h water-deprived rats.

**Discussion:**

We found that WD modulates exploratory activity in rats, but this response is not mediated by oxytocin receptor signaling to the CeA, despite the upregulated Oxtr mRNA expression in that structure after WD for 48 h.

## 1 Introduction

Water and sodium balance in vertebrates involves several neuroendocrine systems that are finely orchestrated to maintain the extracellular fluid (ECF) osmolality and volume within a narrow range of variation. One of the essential responses to this regulation is the development of thirst, which motivates animals to seek and drink water (1). The hypothalamic-neurohypophysial system (HNS) is key to the plasma osmolality control. It is composed of osmosensory magnocellular neurons (MCNs) located at the paraventricular (PVN) and supraoptic (SON) hypothalamic nuclei, and is responsible for producing and secreting the neuropeptides arginine vasopressin (AVP) and oxytocin (OXT) to the blood through neurohypophysis to control kidney function (1). Recent neuroanatomical studies have consistently demonstrated that AVP and OXT MCNs from both PVN and SON send their main axonal projection to the neurohypophysis and also send dense collateral axons to extra-neurohypophyseal brain regions that control anxiety-like behavior, including, among others, the amygdala and lateral habenula (2–5).

Challenges to nutritional homeostasis, such as water deprivation (WD), motivate behaviors related to thirst sensation. Moreover, recent studies of rodents have demonstrated the relationship between body water and sodium balance on the one hand and exploratory behaviors, anxiety and fear response on the other (6–9). Those findings strongly suggest that mechanisms controlling body fluid homeostasis may modulate neuronal circuitries, including the limbic system, related to anxiety and other behavioral responses during dehydration (6). In addition, it has been demonstrated that AVP and OXT MCNs send collateral projections to the basolateral and central amygdala (BLA and CeA, respectively) (4, 5). Furthermore, MCNs AVPergic projections to the lateral habenula (LHb) have also been observed (6). BLA, CeA and LHb have distinct populations of glutamatergic and GABAergic neurons. The balance of their activity is crucial to the final modulation response of those limbic structures at the behavioral level (10, 11). Furthermore, the activation of AVP and OXT receptors produces different physiological responses, with *Avpr1a* inducing an anxiogenic response and *Oxtr* inducing an anxiolytic one (5, 12–14). Hence, it is possible that the imbalance between AVP and OXT contributes to the underlying mechanism of motivated exploratory behaviors in dehydration conditions.

Hyperosmolality is the main stimulus that activates the hypothalamic MCNs, and it can be experimentally induced by WD, inducing both extra- and intracellular dehydration associated with peripheral renin-angiotensin system (RAS) activation, or by salt loading (SL), which induces intra- but not extracellular dehydration, associated with peripheral RAS inhibition (1, 15). Since the RAS has also been found to play an important role in controlling anxiety levels (16), it is possible that the behavioral responses are differentially expressed in WD and SL animals. Therefore, recruitment of RAS, AVPergic and OXTergic systems is an efficient homeostasis regulatory mechanism for coupling hydromineral balance and anxiety-like behaviors.

This study aimed to investigate whether dehydration modulates the exploratory or anxiety-like behaviors at night, the regular activity period of rats. Therefore, we investigated the behavioral responses of rats submitted WD or SL when exposed to the elevated plus maze (EPM) or the open field (OF). We also analyzed *Avpr1a*, *Oxtr*, glutamate decarboxylase 1 (*Gad1*), and vesicular glutamate transporter 2 (*Slc17a6)* mRNA expression in the BLA, CeA and LHb in both hyperosmolality models to elucidate possible plastic molecular responses. Finally, bilateral pharmacological inhibition of OXTR signaling in the CeA was applied to evaluate its potential role in regulating nocturnal exploratory behaviors in rats submitted to WD.

## 2 Materials and methods

### 2.1 Animals

Male Wistar rats (~290g) were obtained from the Animal Facility of the Department of Physiological Sciences, Institute of Biological and Health Sciences, Federal Rural University of Rio de Janeiro (UFRRJ), or from the Center for the Development of Experimental Models for Biology and Medicine (CEDEME) Federal University of São Paulo (UNIFESP). The rats were housed under controlled conditions of temperature (22 ± 2 °C) and 12/12 hours light-dark cycle (lights on at 6 a.m.). All procedures performed were in accordance with current Brazilian legislation and the “Guide for the Care and Use of Laboratory Animals” (17) and were evaluated and approved by the ethical committees for animal use of Federal Rural University of Rio de Janeiro (CEUA-ICBS, protocol number 001/2017) and Federal University of São Paulo (CEUA-UNIFESP, protocol number 7236281119, ID 009443, 2019).

### 2.2 Experimental protocols

**Figure 1:**
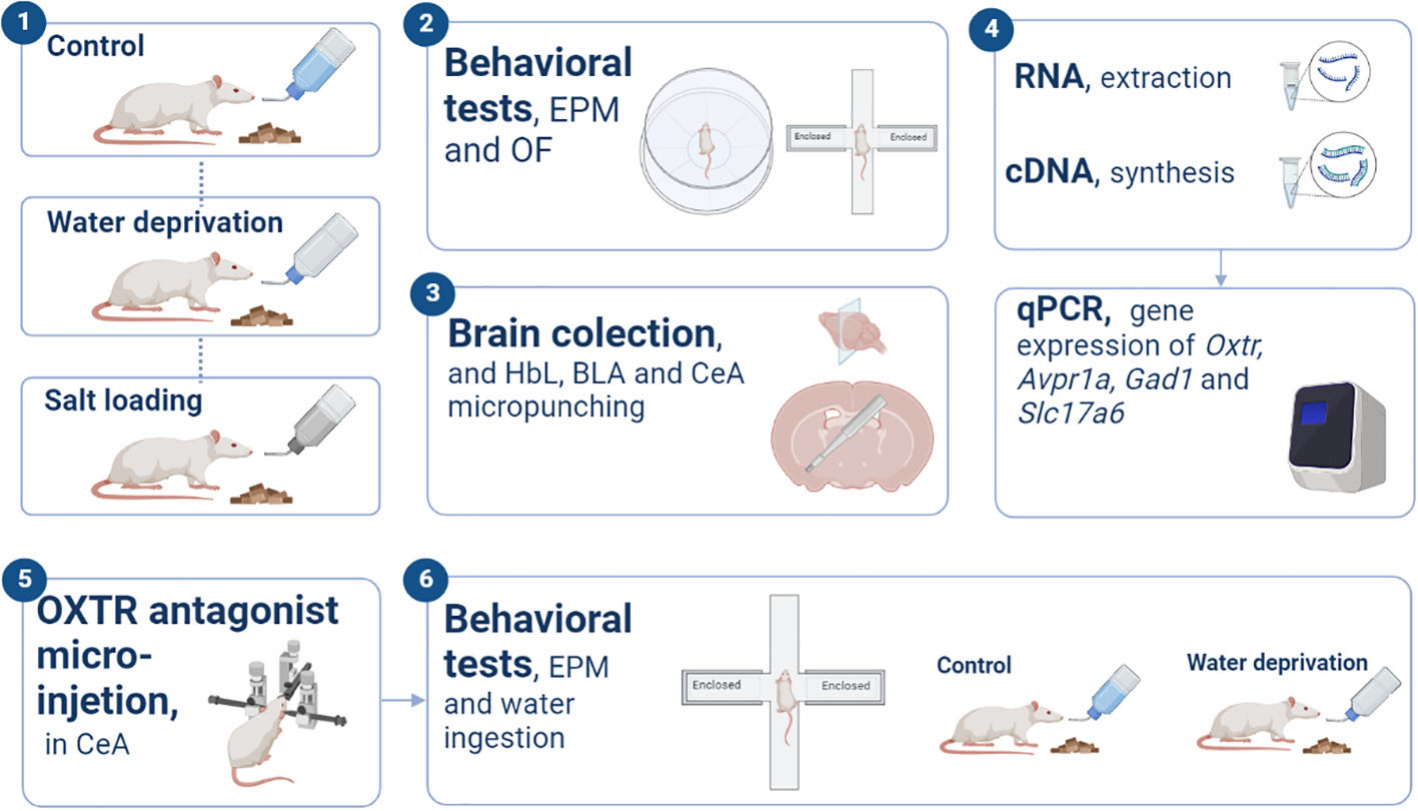
Experimental procedures. Schematic illustration representing the experimental designs and basic procedures employed in the present study. “EPM”, elevated plus maze; “OF”, open field; “LHb”, Lateral habenula; “BLA”, basolateral amygdala; “CeA”, Central amygdala; “OXTR”, oxytocin receptor.

**Figure 1** illustrates the experimental procedures performed in this work. A first set of rats was randomly separated into the following groups: control (CT) which had free access to filtered water and standard chow (1% w/v NaCl, Rhoster, São Paulo, Brazil); WD group, in which water was removed for 24 or 48 hours with free access to standard chow; and SL group, in which the only fluid available was 1.8% NaCl solution for 24 or 48 hours, also with free access to standard chow. The exploratory and anxiety-like behaviors were evaluated *via* 5 minutes of exposure to the elevated plus maze (EPM) test. A different set of animals of control, 48h WD and 48 SL groups were tested for 10 min in the open field test. The behavioral tests were performed at night (7:00 to 11:00 p.m.) because the dark period is the normal activity period of rats (18) and also because Martelli et al., 2012 (9) observed that changes in the locomotor behaviors occur during the dark but not during the light period in water deprived rats. Immediately after the EPM test, the animals were euthanized and blood was collected to determine the hematocrit and plasma osmolality.

A third set of rats was submitted to the control procedure, 48h WD or 48h SL and euthanized at night (7:00 to 11:00 p.m.) for gene expression evaluation by qPCR. The brains were rapidly removed from the skull, frozen with dry ice, and stored at −80°C.

Additionally, to investigate the contribution of OXTR signaling to the WD induced hypoactivity, a fourth set of rats was used to test whether the microinjection of OXTR antagonist in the CeA would be able to alter the dehydration-induced hypoactivity observed in the 48-h WD rats when tested in the EPM. So, 7 days after CeA cannulation, the rats were submitted to a control procedure or 48 h of WD and on day 9 received the OXTR antagonist or vehicle microinjection 30 min before EPM testing. At the end of the test, the rats were returned to their home cages and were allowed to drink water freely. The water consumption, measured in grams, was recorded at 30 and 120 minutes.

### 2.3 Elevated plus maze test

The EPM was employed to analyze the exploratory and anxiety-like behavior in rats (19). This apparatus consists of two opposed open arms (50 × 10 cm each), and two opposed closed arms of the same size with 40 cm high sidewalls, connected by a central area (10 × 10 cm) and raised 50 cm from the ground. Rats were placed into the center area of the apparatus facing a closed arm. Each animal was tested in the EPM for 5 minutes and the apparatus was cleaned between one rat and another with a 10% ethanol solution. The EPM is used to evaluate anxiety-like behavior in rodents. The number of arm entries reflects the locomotion of the animal and the open arm exploration reflects the anxiety-like behaviors. Thus, increased exploration of open arms is associated with decreases in anxiety levels. In addition, the ethological parameter head dipping was evaluated to support the assessment of emotional reactivity. The entries in closed arms and rearing indicate horizontal and vertical exploratory activity, respectively. The time in the center area and the episodes of stretch-attend posture are associated with decision making (19, 20).

### 2.4 Open field test

The open field test was carried out as previously described (21) to confirm that 48 h of WD induced changes in exploratory activity. Each rat was placed individually in the periphery area at the beginning of the test and allowed to explore it freely for 10 min. The arena was cleaned between one rat and another with a 10% ethanol solution. Total, peripheric and central distance travelled and percentage of time spent in the central area were subsequently analyzed using the video-tracking software EthoVision 8.5 (Noldus Information Technology, Leesburg, VA, USA).

### 2.5 Hydromineral parameters

Trunk blood was collected in heparinized tubes and centrifuged (20 min, 3000 rpm at 4 °C), after which plasma osmolality was measured in 10 μl aliquots by using a benchtop osmometer (model 5005, Precision Systems, Natick, MA, USA), based on the freezing-point method. For hematocrit determination, the blood was placed into heparinized capillaries and centrifuged at 2000 rpm for five minutes. Next, a microhematocrit scale was used to determine the percentage of blood composed of erythrocytes.

### 2.6 Microdissection, RNA extraction and cDNA synthesis

Immediately after the EPM test, rats were decapitated and their brains were frozen on dry ice and stored at −80°C. The brains were cut into 60 μm coronal sections using a cryostat (Leica Biosystems, CM 1860, Wetzlar, Germany), and the brain nuclei were identified and delimited according to the rat brain atlas (22). A 1 mm diameter micropunch needle (Fine Science Tools) was used to bilaterally collect LHb (coordinates from Bregma −2.30 to −4.70 mm), BLA (coordinated from Bregma −1.80 to −3.80 mm) and CeA (coordinated from Bregma -1.80 to -3.30 mm). The micropunch material was immediately placed in microtubes containing TRIzol^®^ reagent (Life Technologies, Waltham, MA, USA) and stored at −80°C. Sections were mounted on glass slides and stained with 0.1% toluidine blue to confirm the location of the punches using a light microscope. Micropunches from incorrect dissection were not used. Total RNA was extracted from punch samples using TRIzol^®^ reagent as recommended by the manufacturer, and was quantified in a nanospectrophotometer (DS-11^®^ Denovix Inc. Wilmington, DE, USA). Samples presenting a ratio of optical density (OD) 260/280 in the 1.8-2.1 range were used for reverse transcription. The synthesis of cDNA was performed using the QuantiTect Reverse Transcription Kit (Qiagen) with 500 ng of total RNA. The cDNA obtained was diluted in a proportion of 1:3 and stored at –20°C.

### 2.7 Quantitative real-time polymerase chain reaction

The qPCR was performed in duplicate or triplicate using SYBR green (Applied Biosystems) or Taqman Universal PCR Master Mix kit (Carlsbad, CA, USA). The qPCR was performed with the QuantStudio 3^®^ system (ThermoFisher Scientific). With the SYBR green, the following primers (Applied Biosystems) were used: *Avpr1a* (5′-ATCTGCTACCACATCTGGCG-3′ and 5′-TTATGAAAGGGACCCACGGC-3′), *Oxtr* (5′-CTTCATCCAACCCTGGGGAC-3′ and 5′-CTTGAAGCTGATGAGGCCG–3′), and *Rpl19* (5′-GCGTCTGCAGCCATGAGTA-3′ and 5′-TGGCATTGGCGATTTCGTTG-3′) as the endogenous control gene. With the Taqman Gene Expression Assays (Applied Biosystems), the following probes were used: *Gad1* (Rn00690300_m1), *Slc17a6* (Rn00584780_m1), and *Actb* (Rn00667869_m1) as endogenous control. The reactions were performed using an ABI 7500 Sequence Detection System (ABI, Warrington, UK), with universal cycling conditions carried out according to the manufacturer’s instructions. The genes RPL19 and β-Actin give highly reproducible and stable expression measurements between different physiological challenges within the CeA, BLA and LHb. For relative quantification of gene expression, the 2^−ΔΔCT^ method was employed (23).

### 2.8 The OXTR antagonist microinjection in the CeA

Rats were anesthetized with ketamine (100 mg/Kg; i.p.) and xylazine (10 mg/Kg; i.p.). After the onset of anesthesia, animals received ketoprofen (3 mg/kg; s.c.) as analgesic, pentobiotic (30000 I.U./Kg; i.m.) as antibiotic, polyacrylic acid as eye lubricant and lidocaine 0.5% as local anesthetic in ears and at the incision site. A 23 G and 14 mm length cannula was implanted bilaterally using the following stereotaxic coordinates relative to the bregma: posterior −2.2 mm, lateral ± 4.3 mm and ventral −7.6 mm. A stylet was inserted into the guide cannula for obturation. The cannula guide and the stylet were fixed with dental cement. Twenty-four hours after surgery, the ketoprofen injection was repeated. Rats were allowed to recover for one week after the surgical procedure. Two hours before the EPM test, rats were individually placed in standard polyethylene cages and the stylet was removed from the guide cannula. Thirty minutes before the EPM test, a 32 G needle connected to a polyethylene tube (PE 10), which in turn was connected to a 1 µL syringe (Hamilton) was inserted and extended 0.2 mm from the end of the cannula guide. Then, 0.4 µl of the OXTR antagonist L-371,257 (Santa Cruz Biotechnology, Inc., Dallas, TX, 1; sc-204038; 4 µM in 0.9% saline-10% DMSO) or vehicle (0.9% saline-10% DMSO) was manually infused during 1 min. The needle was left for 1 additional minute to allow the complete diffusion of the drug and then it was removed. The antagonist dose was chosen based on the literature (4, 24).

In order to verify the right infusion site at the CeA (**Supplementary Figures 1** and **2**), at the end of the experiment the rats were administrated identically as described above with 0.4 µl of 0.2% Evans blue, anesthetized with isoflurane and decapitated. Then the brains were removed and fixed by placing them into a solution of 4% paraformaldehyde in 0.1 M phosphate buffer for two days. Fixed brains were sliced into 60 µm coronal sections with a cryostat (Leica Microsystems CM1850 Cryostat; Wetzlar, Germany) and light microscopy was used to confirm the infusion site according to the Paxinos atlas (22). Data from rats with one or two misplaced cannulas were not included in the analyses.

### 2.9 Statistical analysis

All values are presented as means ± SD. Data from hematocrit, plasma osmolarity, EPM, OF and qPCR with normal distribution (depending on the Shapiro–Wilk test) were subjected to 1-way analysis of variance (ANOVA; the value of the F-statistic is reported) with 3 levels: control, WD and SL. Significant differences between groups were further analyzed through the Tukey *post hoc* test. When these variables were not normally distributed, data were subjected to the Kruskal-Wallis test (the value of the H-statistic is reported) with three levels: control, WD and SL. Significant differences between groups were further analyzed through Dunn’s multiple comparisons test. Data from OXTR antagonist were submitted to 2-way ANOVA with the hydration status factor having two levels (control and WD) and the drug administration factor also with two levels (vehicle or OXTR antagonist). The values of stretch-attend posture and rearing in EPM did not have normal distribution and were rank transformed before statistical analysis according to Hora and Conover, 1984 (25). All statistical analyses were conducted with the GraphPad Prism software (version 8, San Diego, USA). In all cases, p-values smaller than 0.05 were considered to indicate a significant effect.

## 3 Results

### 3.1 Effects of dehydration on the plasma osmolality and hematocrit

**Figure 2:**
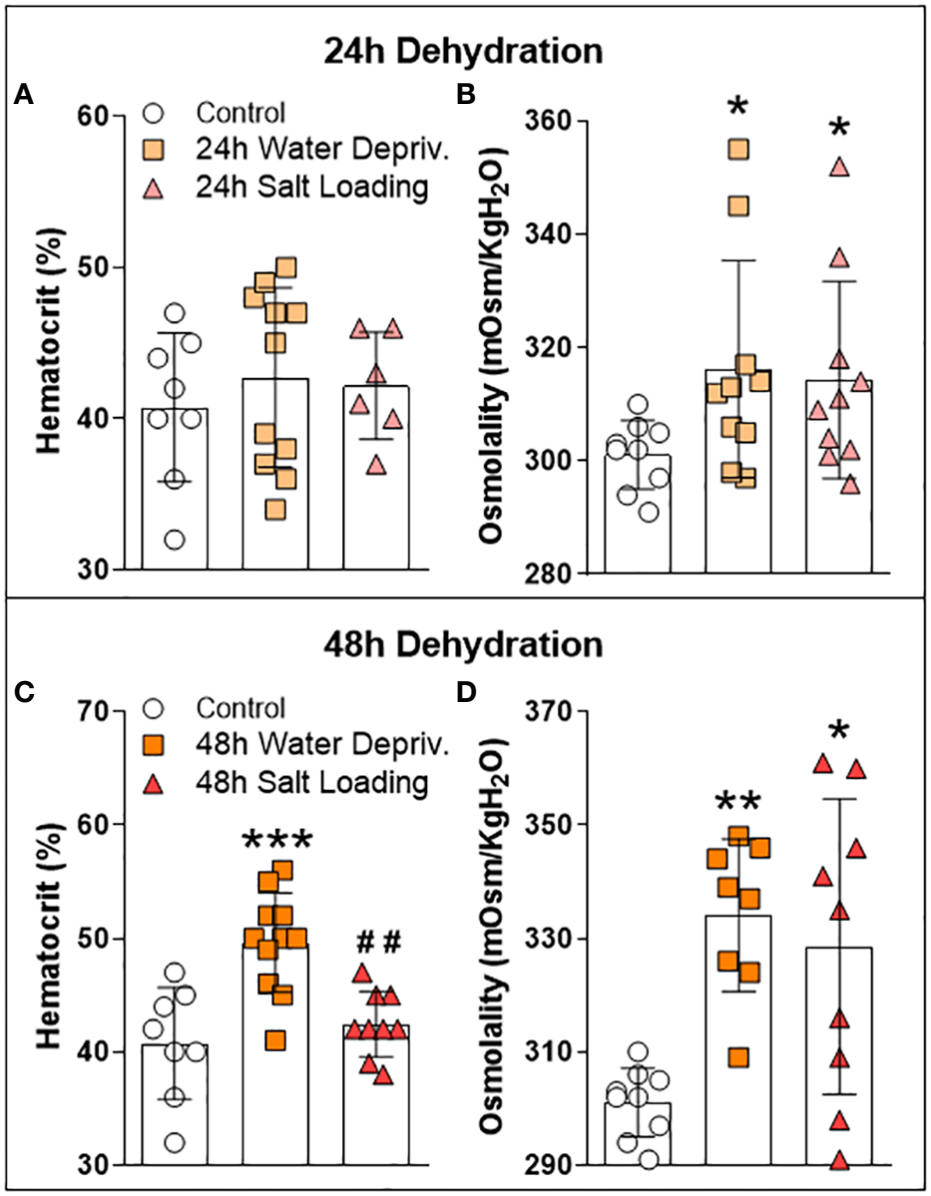
Effects of **(A**, **B)** 24 h or **(C**, **D)** 48 h of dehydration in male adult rats on **(A**, **C)** hematocrit and **(B**, **D)** plasma osmolality. Values are mean ± SD. The number of animals used per group was: Control = 9; 24 h WD = 11; 24 h SL = 10; 48 h WD = 11; 48 h SL = 9. Hematocrit data were submitted to one-way ANOVA followed by the Tukey post hoc test. Osmolality data were analyzed by the Kruskal-Wallis test followed by Dunn’s post hoc test. *p<0.05, **p<0.01 and ***p<0.001 compared to control groups; ##p<0.01 compared to the WD group.

In order to validate our dehydration models (WD and SL), we evaluated the plasma osmolality and the hematocrit (**Figure 2** and **Supplementary Table 1**). As expected, osmolality was significantly affected by dehydration protocols after 24 [H=6.051; df=2; p=0.0485; **Figure 2B**] and 48 hours [H=10.94; df=2; p=0.0042; **Figure 2D**]. The hematocrit was affected by 48 h of WD [F_(2,25)_=12.86; p=0.0001] since rats submitted to 48 h of WD showed higher hematocrit values than the control and the SL groups (p=0.0003 and p=0.0019; respectively; **Figure 2C**). These results demonstrated that after 48 h, both WD and SL increased the plasma osmolality, thus validating our dehydration models.

### 3.2 Effects of WD and SL on the anxiety-like and locomotory behaviors

**Figure 3:**
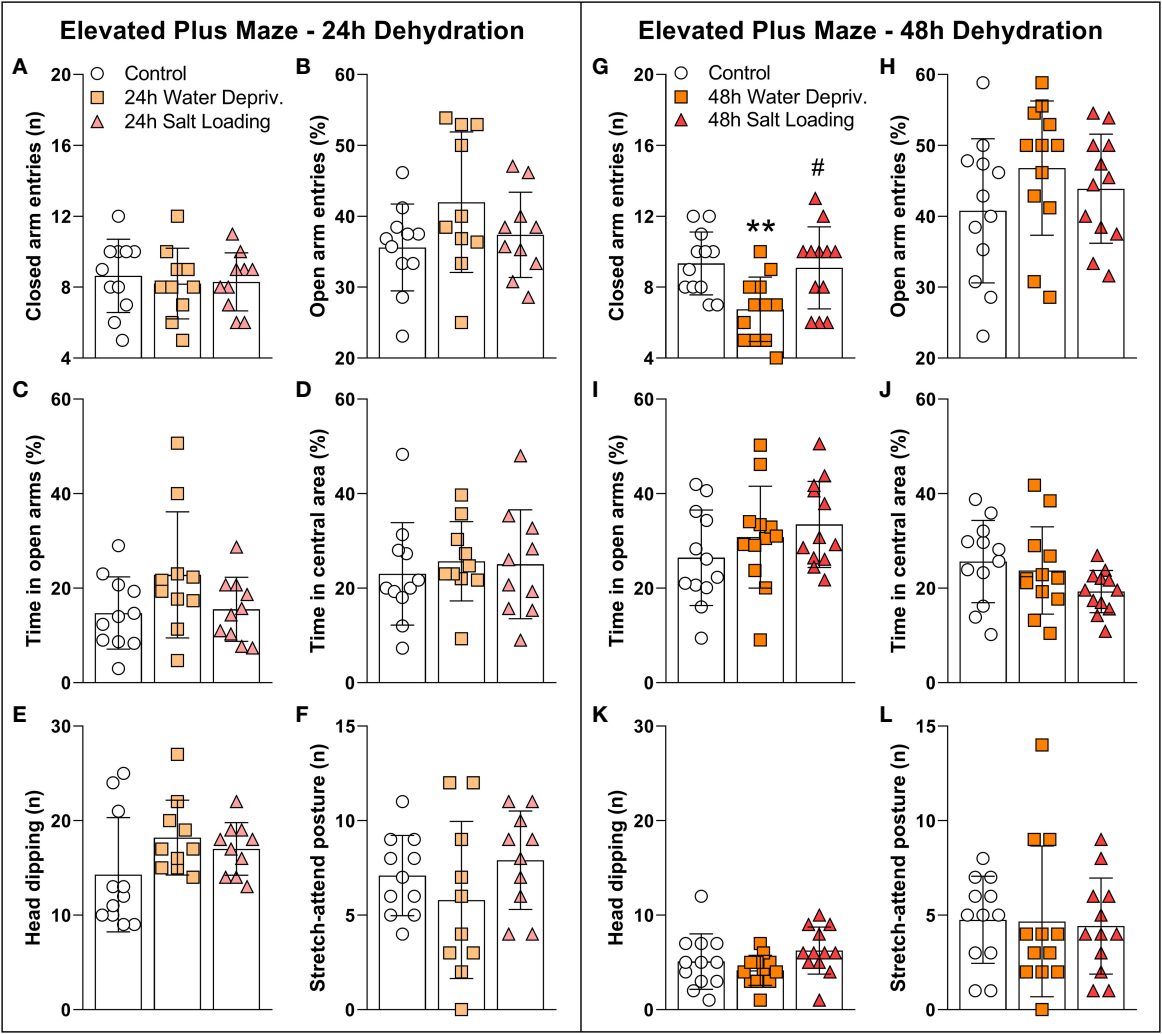
Effects of **(A–F)** 24 h or **(G–L)** 48 h of dehydration in male adult rats on **(A, G)** number of entries into closed arms, **(B, H)** percentage of entries into open arms, **(C, I)** percentage of time spent in open arms, **(D, J)** percentage of time spent in the central area, **(E, K)** number of head dipping episodes, and **(F, L)** number of stretch-attend postures during 5 min of evaluation in the elevated plus maze apparatus. Values are mean ± SD. The number of animals used per group was: 24 h Control = 11; 24 h WD = 10; 24 h SL = 10; 48 h Control = 12; 48 h WD = 12; 48 h SL = 12. Data were submitted to one-way ANOVA followed by the Tukey *post hoc* test, except for the percentage of time spent in the open arms after 24 h of dehydration and the number of stretch-attend postures after 48 h of dehydration, in which the Kruskal-Wallis test was used. **p<0.01 compared to control group; ^#^p<0.05 compared to the WD group.

We carried out the EPM test to assess whether dehydration triggered by WD and SL influences anxiety-like behavior and/or locomotion (**Figure 3** and **Supplementary Table 2**). **Figure 3A–F** shows that 24 h of WD or SL did not alter rats’ behavior in the EPM test. However, after 48 h of dehydration (**Figure 3G**), we found a significant effect on closed arm entries [F_(2,33)_=6.192; p=0.0052], with WD rats entering less in the closed arms than the control (p=0.0085) and SL rats (p=0.0185). The other parameters assessed were not altered by 48 h of WD or SL (**Figures 3G–L**). These results suggest that 48 h of WD induces hypolocomotion in EPM without affecting anxiety-like behavior.

**Figure 4:**
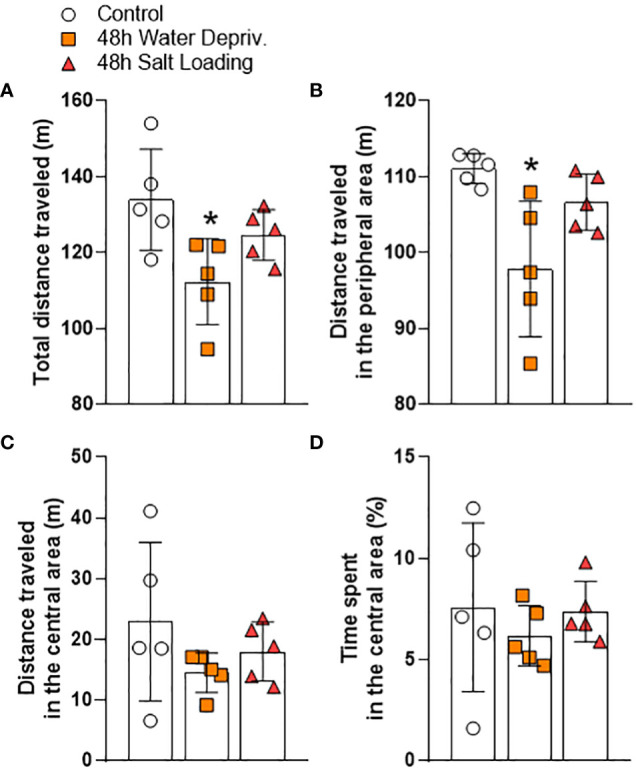
Effects of 48 h of dehydration in male adult rats on **(A)** total locomotion, **(B)** locomotion in the peripheral area, **(C)** locomotion in the central area, and **(D)** percentage the time spent in the central area during 10 min of evaluation in the open field test. Values are mean ± SD of 5 animals per group. Data were submitted to one-way ANOVA followed by the Tukey *post hoc* test, except for the distance traveled in the peripheral area, in which the Kruskal-Wallis test was used, followed by Dunn’s *post hoc* test. *p<0.05 compared to the control group.

Additionally, the open field test (**Figure 4** and **Supplementary Table 3**) showed a decrease in total (**Figure 4A**) and peripheral (**Figure 4B**) exploration of 48 h WD group [F_(2,12)_=5.05; p=0.0256 and H=8.42; p=0.0068; d.f. = 12; respectively]. On the other hand, the distance traveled and time spent in the central area were not affected by WD or SL, confirming that 48 h reduced nocturnal exploratory behavior without significantly affecting the anxiety-like behaviors.

### 3.3 Effects of WD and SL on the gene expression in LHb, BLA, and CeA

**Figure 5:**
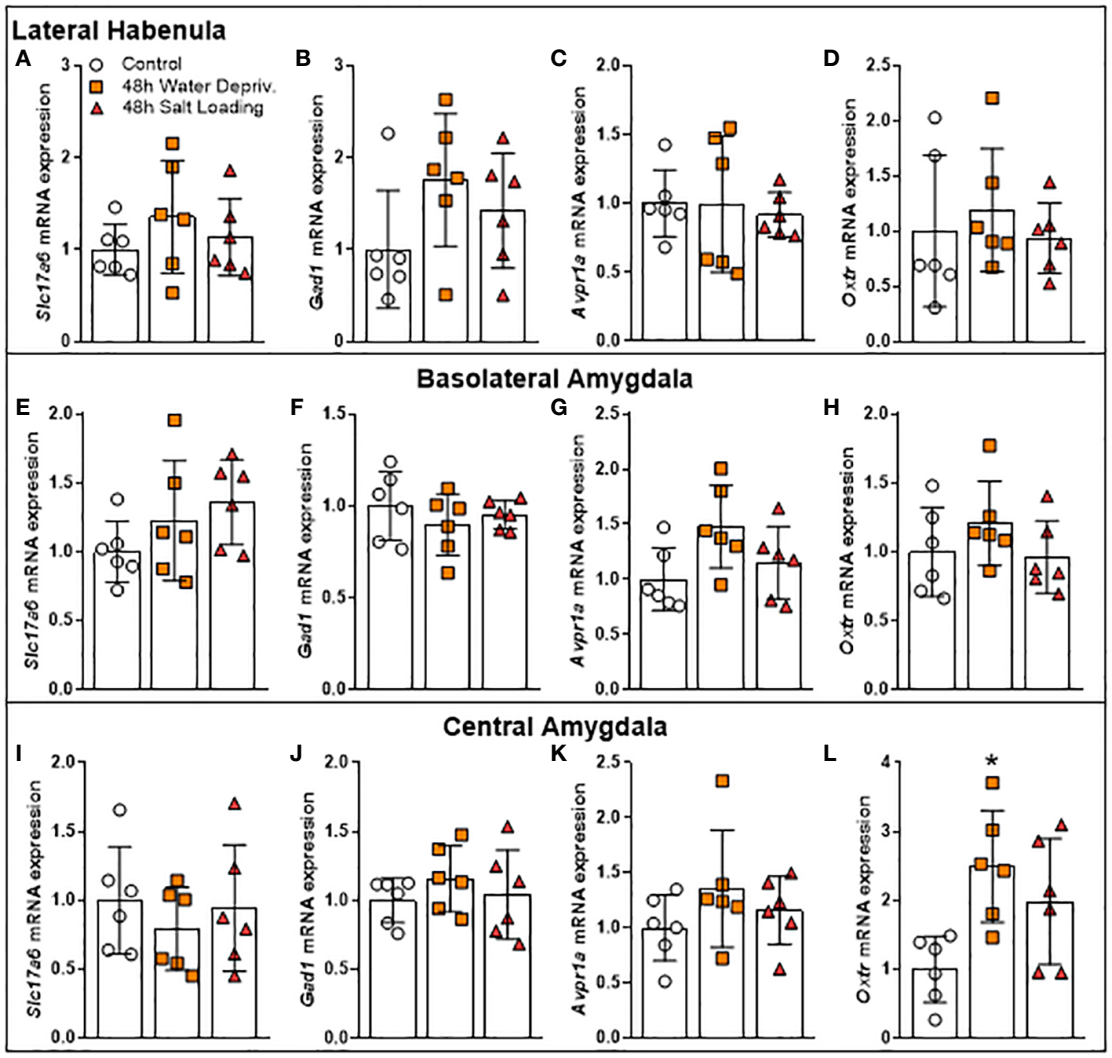
Effects of 48 h of dehydration in male adult rats on relative mRNA expression of **(A, E, I)** the Vesicular Glutamate Transporter 2 (*Slc17a6*), **(B, F, J)** the Glutamate Decarboxylase 1 (*Gad1*), **(C, G, K)** the Arginine Vasopressin Receptor 1A (*Avpr1a*), and **(D, H, L)** the Oxytocin Receptor (*Oxtr*) in the **(A–D)** lateral habenula, **(E–H)** the basolateral amygdala (BLA), and **(I-L)** the central amygdala (CeA). Values are mean ± SD of 6 animals per group. Data of gene expression in BLA as well as *Oxtr* mRNA expression in CeA were analyzed by one-way ANOVA followed by the Tukey *post hoc* test. The other data were submitted to the Kruskal-Wallis test. *p<0.05 compared to the control group.

Dehydration activates the MCNs, increasing AVP and OXT synthesis and secretion (1). In addition to their classical osmoregulation function, these neuropeptides have key roles in modulating anxiety-like and locomotory behaviors (6, 9). For this reason, we investigated the gene expression of AVP and OXT receptors in the CeA, BLA and LHb, limbic structures known to regulate anxiety-like and locomotory behaviors and to receive projections from MCNs (2, 5, 6). Additionally, we measured the mRNA expression of gene markers of GABAergic (Gad1) or glutamatergic (Slc17a6) neurons to infer whether the dehydration could modulate the level of excitation or inhibition of the neurons of the structures mentioned above (**Figure 5** and **Supplementary Table 4**).

While dehydration did not change the expression of these genes in the LHb (**Figures 5A–D**) and BLA (**Figures 5E–H**), it altered *Oxtr* gene expression in CeA [F_(2,15)_=5.994; p=0.0122]. Our data showed that rats submitted to 48 h of WD had higher *Oxtr* gene expression levels in CeA compared to controls (p=0.0103; **Figure 5L**). The gene expression values of *Slc17a6*, *Gad1* and *Avpr1a* in CeA were not altered by the dehydration model (**Figures 5I–K**). Thus, we investigated whether OXTR in CeA can modulate the locomotor response of rats.

### 3.4 Effect of OXTR antagonist microinjection in CeA

**Figure 6:**
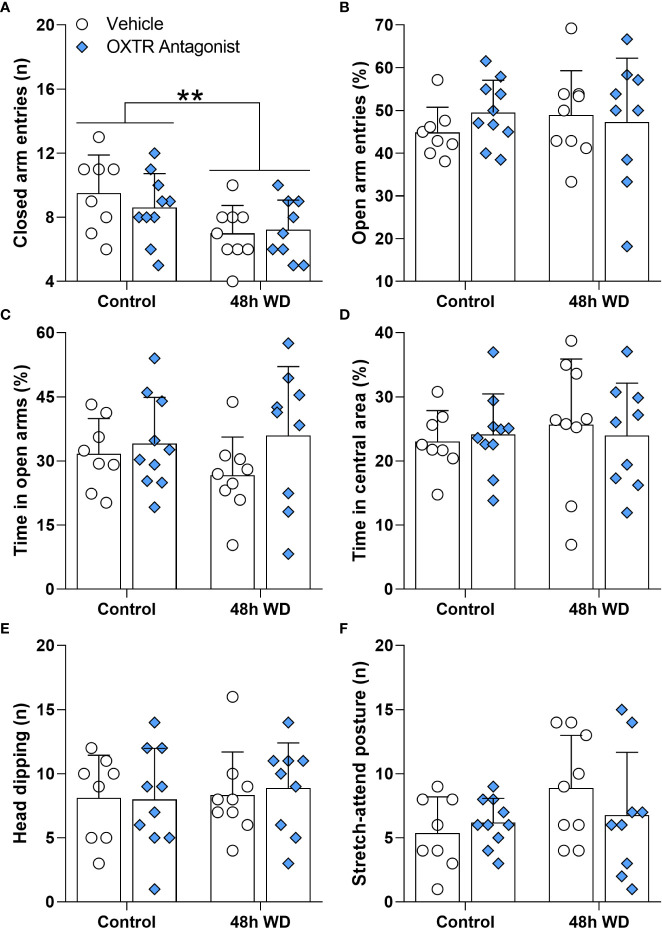
Effects of oxytocin receptor antagonist microinjection in the central amygdala of 48-h water-deprived male adult rats on **(A)** number of entries into closed arms, **(B)** percentage of entries into open arms, **(C)** percentage of time spent in open arms, **(D)** percentage of time spent in the central area, **(E)** number of head dipping episodes, and **(F)** number of stretch-attend postures during 5 min of evaluation in the elevated plus maze apparatus. Values are mean ± SD. The number of animals used per group was: Control + vehicle = 8; Control + antagonist = 10; 48 h WD + vehicle = 9; 48 h WD + antagonist = 9. Data were submitted to two-way ANOVA. The values of the number of stretch-attend postures were transformed to ranks before ANOVA. **p<0.01 comparing Water Deprived *vs*. Control groups.

To further investigate whether OXTR signaling in the CeA mediates the behavioral alteration observed in WD rats, we injected the OXTR antagonist L-371,257 into the CeA of rats after 48 h of WD. Twenty minutes later, the animals were submitted to the EPM test (**Figure 6** and **Supplementary Table 5**). **Figure 6A** shows that rats submitted to 48 h of WD entered the closed arms less than the control rats (not water deprived) [F_(1,32)_=8.154; p=0.0075]. However, the OXTR antagonist used failed to reverse the reduction of closed arm exploration in water-deprived rats. Open arm entries and time in the center area, head dipping and stretch-attend posture episodes (**Figures 6B–F**) were not affected by the OXTR antagonist or by WD. Note that these data match those presented in **Figure 3**. There was a significant effect of antagonist administration on reduction of rearing episodes [F_(1,32)_=5.670; p=0.0234], demonstrating the efficiency of the oxytocin receptor antagonist used (**Supplementary Figure 3** and **Supplementary table 5**).

**Figure 7:**
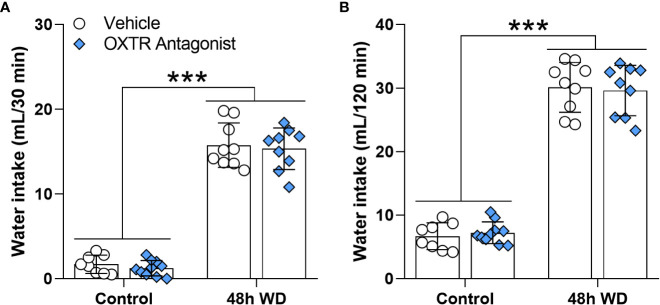
Effects of oxytocin receptor antagonist microinjection in the central amygdala of 48 h water-deprived male adult rats on water intake during **(A)** 30 and **(B)** 120 minutes. Values are mean ± SD. The number of animals used per group was: Control + vehicle = 8; Control + antagonist = 10; 48 h WD + vehicle = 9; 48 h WD + antagonist = 9. Data were submitted to two-way ANOVA.

At the end of the plus maze test, animals were allowed to drink water freely, and the respective intakes at 30 and 120 minutes (**Figures 7A, B** and **Supplementary Table 6**) were recorded. As expected, water-deprived rats drank more water than the control animals at 30 and at 120 minutes [F_(1,33)_=504.8; p<0.0001 and F_(1,33)_=521.4; p<0.0001; respectively]. However, the oxytocin receptor antagonist had no effect on the water drank by the rats (**Figures 7A, B**).

## 4 Discussion

It has been suggested that homeostatic signals, such as thirst, can modulate emotion, motivation, and motor functions (6). Here we used two dehydration models to induce thirst and activate the MCNs of PVN and SON: WD and SL (1). As we expected, both protocols increased plasma osmolarity, agreeing with previous results using mice (26) and rats (27), and validating our models. Additionally, WD increased the hematocrit, as was also expected, coinciding with the previously mentioned results of Fujio et al., 2006 (27).

Our results support the notion that locomotor alterations occur in response to WD. A previous study (9) showed a decrease in locomotion of water-deprived rats within their home cage only during the dark period. Our results corroborate and extend those of Martelli et al., 2012 (9), since we demonstrated that locomotion is also impaired in novel environments. Interestingly, hyperosmotic thirst induced by SL does not produce alterations in locomotion even though both kinds of thirst activate hypothalamic MCNs (1, 28, 29). Although both models induce dehydration, they have several differences (29). While plasma osmolality is progressively increased following WD and SL, urinary osmolality only increases with WD. In addition, WD reduces food intake by approximately 50% compared to SL. AVP and OXT levels are increased in both models compared to controls. However, the AVP level is lower, but the OXT level is higher in the WD compared to SL. It is likely that the physiological and behavioral discrepancies in response to WD and SL result from different central activation of several brain nuclei in each model. Indeed, transcriptome analysis of SON after SL or WD revealed 7060 genes are regulated by WD but not by SL (29). On the other hand, the anxiety-like behavior evaluated in the EPM was not affected by any of the thirst models used. In contrast, Zhang et al. (2016) found a decrease in the open arm exploration after 24 h of WD (6). In that work, rats were evaluated during the light period of the light/dark cycle. Probably evaluating animals during the normally non-active period and under an aversive stimulus such as light affected their behavior, explaining the differences with our results.

In the cited work of Zhang et al. (2016), increased activation (assessed by Fos-immunoreactivity) of GABAergic neurons of the LHb was found (6). The authors suggested that the habenula may link the forebrain with midbrain structures that regulate emotional behavior, since habenula lesions resulted in stress, anxiety, reward and motor dysfunctions (30). For example, habenular lesions prompted animals to increase anxiety-like behavior in the EPM test and increased locomotion in the open field test (31, 32). The amygdala participates in the fluid intake regulation (33). Additionally, it has already been shown that WD increases Fos expression in the CeA (34) and it is well established that BLA (35, 36) and CeA (37, 38) mediate anxiety-like behavior. However, our data show that *Gad1*, *Slc17a6, Avpr1a a*nd *Oxtr* mRNA expression are not changed in LHb or BLA after 48 h of WD or SL. Therefore, the observed changes found in the locomotion of rats submitted to 48 h of WD were not mediated by expression levels of these genes in the LHb or BLA. However, we cannot discard that the protein expression and/or post-transcriptional modifications may result in functional alterations of oxytocin and/or vasopressin type 1a receptors, as well as glutamatergic and/or GABAergic signaling in those brain areas.

Regarding the CeA, we found an increase in the *Oxtr* mRNA expression. This brain structure has a large number of oxytocin receptors involved, among others, in fear behavior inhibition (2, 39) and stress-coping behavior (40). Moreover, subcutaneous (41) and intracerebroventricular (42) oxytocin administration reduces anxiety-like behavior in rats. Also, Windle et al., 1997 (42) reported a reduction of rearing by i.c.v. administration of oxytocin. Since the CeA has oxytocin receptors, whose activation regulates several behaviors, we investigated the possibility that OXT acting on the CeA could regulate locomotion in rats. To determine whether oxytocin receptors were involved in the locomotor alteration of water-deprived rats observed in the EPM, we administrated an oxytocin receptor antagonist in the CeA. The results showed that the OXTR antagonist did not reverse the exploratory reduction of 48-h water-deprived rats or change water intake of those rats. On the other hand, the OXTR antagonist decreased vertical exploration (assessed as rearing episodes in the EPM test) independently of the hydration state of the animal. In addition to demonstrating that oxytocin receptor blockade was efficient, this result indicates that although the general action of oxytocin on the central nervous system can decrease exploration (41, 43), the activation of CeA oxytocin receptors seems to stimulate exploration, since their blocked reduced rearing. On the other hand, the WD-induced alteration in locomotory activity may involve other brain nuclei. In the present work, we focus on limbic structures involved in anxiety-like and exploratory behaviors. In future works, it would be interesting to study gene expression in brain nuclei that control voluntary motricity. Of particular interest may be the caudate-putamen, a mesolimbic structure involved in motor activity and motivation behaviors (44), which also participates in thirst regulation (45) and expresses oxytocin receptors (46).

The present study provides evidence that WD modulates the exploratory activity and upregulates *Oxtr* expression in the CeA. However, our work has some limitations to consider. First, to dissolve the OXT antagonist, we had to use 10% DMSO. This compound can affect the excitability of neurons, having a considerable inhibitory effect (47). Although the antagonist-treated groups were compared to vehicle-treated groups that also received 10% DMSO, future works using other antagonists or solvents are needed. Secondly, this work would benefit from others that confirm the changes observed here in the *Oxtr* expression through different techniques, such as RNAscope, to add information on the cellular location of the *Oxtr* whose expression was altered. Finally, considering that plasma levels of angiotensin II are increased by WD and decreased by SL (29), it might be interesting to study the gene expression of the RAS components in the brain areas regulating exploratory behavior, such as the amygdaloid complex. It has been found that the microinjection of a selective AT1 antagonist into the amygdala induces an anxiolytic-like effect in rats, increasing the time spent in the EPM open arms and the total of entries (48). On the other hand, the microinjection of PD123319, a selective AT_2_ antagonist, into the medial amygdala of rats increased anxiety-like behavior assessed in EPM (49). These data indicate that the differential levels of angiotensin II at the circulation or in the brain, or even changes in its signaling mechanisms, might contribute to locomotor activity changes observed in rats submitted to WD but not to SL. Thus, future studies might address the role of the RAS in the nocturnal hypoactivity induced by WD in rats.

In summary, we found dehydration-induced hypoactivity and increased levels of *Oxtr* mRNA expression in the CeA after 48 h of WD. However, after blockade of the OXTR signaling to the CeA of WD rats, we found no significant changes on the nocturnal exploration in the EPM, indicating that OXTR signaling to the CeA does not mediate dehydration-induced hypoactivity in male rats.

## Data availability statement

The raw data supporting the conclusions of this article will be made available by the authors, without undue reservation.

## Ethics statement

The animal study was reviewed and approved by ethical committees for animal use of Federal Rural University of Rio de Janeiro (CEUA-ICBS, protocol number 001/2017) and Federal University of São Paulo (CEUA-UNIFESP, protocol number 7236281119, ID 009443, 2019).

## Author contributions

AM, LR, and FR contributed to the conception and design of the study. AM supervised the project and acquired financial support. VF, VT, RD-S, CA, and AM performed the experiments. VF, VT, and AM analyzed the data. VF and VT wrote the first draft of the manuscript. All authors contributed to manuscript revision, read, and approved the submitted version.

## Supplementary material

Supplementary Figure 1 Histological location at −2.16 mm posterior to bregma of the cannulas implanted in CeA are shown for control rats administered with vehicle (blue circles) or OXTR antagonist (green circles) and 48-h water-deprived rats administered with vehicle (pink circles) or OXTR antagonist (violet circles).

Supplementary Figure 2 Drawing of a coronal section of rat brain at bregma -2.16 mm, and representative microphotograph at 4x showing the microinjection site in the central amygdala. “CeA”, central amygdala; “opt”, optic tract; “ec”, external capsule; and “I.S.”, injection site.

Supplementary Figure 3 Effects of oxytocin receptor antagonist microinjection in the central amygdala of 48-h water-deprived male adult rats on rearing episodes during 5 min of evaluation in the elevated plus maze apparatus. Values are mean ± SD. The number of animals used per group was: Control + vehicle = 8; Control + antagonist = 10; 48 h WD + vehicle = 9; 48 h WD + antagonist = 9. Data were submitted to two-way ANOVA. The values were transformed to ranks before submission to two-way ANOVA. *p<0.05 comparing rats receiving OXTR antagonist *vs*. rats receiving vehicle.
