# Supplementary material for: Water deprivation induces hypoactivity in rats independently of oxytocin receptor signaling at the central amygdala
Source: Front Endocrinol (Lausanne). 2023 Jan 31;14:1062211. doi: 10.3389/fendo.2023.1062211 (PMC9928579; doi:10.3389/fendo.2023.1062211)
Supplement: Supplementary file 1 [file Table_1.docx]

**Supplementary Table 1**. Hematocrit and plasma osmolality

|  | **Control** | **WD** | **SL** | **Statistics** |
| --- | --- | --- | --- | --- |
| **24h** |  |  |  |  |
| Hematocrit (%) | 40.8 ± 4.9, n=8 | 42.7 ± 5.9, n=11 | 42.2 ± 3.5, n=6 | F_(2,22)_= 0.347, p=0.711 |
| Osmolality (mOsm/KgH_2_O) | 301.1 ± 6.1, n=9 | 316.2 ± 19.1 *, n=10 | 314.3 ± 17.4 *, n=10 | H= 6.051, p=0.049, d.f.=26 |
| **48h** |  |  |  |  |
| Hematocrit (%) | 40.8 ± 4.9, n=8 | 49.6 ± 4.4 ***, n=11 | 42.4 ± 2.9 ^# #^, n=9 | F_(2,25)_=12.86, p<0.001 |
| Osmolality (mOsm/KgH_2_O) | 301.1 ± 6.1, n=9 | 334.1 ± 13.4 **, n=8 | 328.6 ± 26.0 *, n=9 | H= 10.94, p=0.004, d.f.=23 |

Effects of 24 h or 48 h of water deprivation (WD) or salt loading (SL) on hematocrit and plasma osmolality in male adult rats. Values are mean ± SD. Hematocrit data were submitted to one-way ANOVA followed by the Tukey post hoc test. Osmolality data were analyzed by the Kruskal-Wallis test, followed by Dunn’s post hoc test. *p<0.05, ***p<0.01 and ***p<0.001 compared to control groups; ##p<0.01 compared to WD group.
