# Supplementary material for: Water deprivation induces hypoactivity in rats independently of oxytocin receptor signaling at the central amygdala
Source: Front Endocrinol (Lausanne). 2023 Jan 31;14:1062211. doi: 10.3389/fendo.2023.1062211 (PMC9928579; doi:10.3389/fendo.2023.1062211)
Supplement: Supplementary file 2 [file Table_2.docx]

**Supplementary Table 2**. Elevated plus maze test

|  | **Control** | **WD** | **SL** | **Statistics** |
| --- | --- | --- | --- | --- |
| **24h** | n=11 | n=10 | n=10 |  |
| Closed arm entries (n) | 8.64 ± 2.06 | 8.20 ± 1.99 | 8.30 ± 1.64 | F_(2,28)_= 0.152, p=0.860 |
| Open arm entries (%) | 35.6 ± 6.1 | 42.0 ± 9.9 | 37.4 ± 6.0 | F_(2,28)_= 1.972, p=0.158 |
| Time in open arms (%) | 14.7 ± 7.6 | 22.8 ± 13.3 | 15.5 ± 6.8 | H= 3.011, p=0.222, d.f.=28 |
| Time in central area (%) | 23.0 ± 10.9 | 25.7 ± 8.4 | 25.0 ± 11.5 | F_(2,28)_= 0.189, p=0.829 |
| Head dipping (n) | 14.3 ± 6.1 | 18.2 ± 4.0 | 17.0 ± 2.8 | F_(2,28)_= 2.078, p=0.144 |
| Stretch-attend posture (n) | 7.09 ± 2.12 | 5.80 ± 4.16 | 7.90 ± 2.60 | F_(2,28)_= 1.203, p=0.315 |
| **48h** | n=12 | n=12 | n=12 |  |
| Closed arm entries (n) | 9.33 ± 1.78 | 6.75 ± 1.82 ** | 9.08 ± 2.31 ^#^ | F_(2,33)_= 6.192, p=0.005 |
| Open arm entries (n) | 40.8 ± 10.2 | 46.8 ± 9.5 | 43.9 ± 7.7 | F_(2,33)_= 1.293, p=0.288 |
| Time in open arms (%) | 26.4 ± 10.1 | 30.8 ± 10.8 | 33.5 ± 9.1 | F_(2,33)_= 1.514, p=0.235 |
| Time in central area (%) | 25.6 ± 8.7 | 23.7 ± 9.2 | 19.3 ± 4.5 | F_(2,33)_= 2.117, p=0.136 |
| Head dipping (n) | 5.08 ± 2.94 | 4.17 ± 1.59 | 6.25 ± 2.49 | F_(2,33)_= 2.262, p=0.120 |
| Stretch-attend posture (n) | 4.75 ± 2.30 | 4.67 ± 3.99 | 4.42 ± 2.54 | H= 0.554, p=0.758, d.f.=33 |

Effects of 24 h or 48 h of water deprivation (WD) or salt loading (SL) in male adult rats on ethological and exploratory parameters assessed during 5 min in the elevated plus maze test. Values are mean ± SD. Data were submitted to one-way ANOVA followed by the Tukey post hoc test, except for the percentage of time spent in the open arms after 24 h of dehydration and the number of stretch-attend postures after 48 h of dehydration, in which the Kruskal-Wallis test was used. **p<0.01 compared to control group; #p<0.05 compared to WD group.
