# Supplementary material for: Water deprivation induces hypoactivity in rats independently of oxytocin receptor signaling at the central amygdala
Source: Front Endocrinol (Lausanne). 2023 Jan 31;14:1062211. doi: 10.3389/fendo.2023.1062211 (PMC9928579; doi:10.3389/fendo.2023.1062211)
Supplement: Supplementary file 3 [file Table_3.docx]

**Supplementary Table 3**. Open field test

|  | **Control (n=5)** | **48h WD (n=5)** | **48h SL (n=5)** | **Statistics** |
| --- | --- | --- | --- | --- |
| Total distance traveled (m) | 133.9 ± 13.3 | 112.3 ± 11.3 * | 124.6 ± 6.7 | F_(2,12)_= 5.053, p=0.026 |
| Distance traveled in the peripheral area (m) | 111.1 ± 2.0 | 97.8 ± 8.9 * | 106.6 ± 3.7 | H= 8.420, p=0.0068, d.f.=12 |
| Distance traveled in the central area (m) | 22.9 ± 13.1 | 14.5 ± 3.2 | 18.0 ± 4.8 | F_(2,12)_= 1.306, p=0.307 |
| Time spent in the central area (%) | 7.58 ± 4.17 | 6.17 ± 1.48 | 7.37 ± 1.49 | F_(2,12)_= 0.394, p=0.683 |

Effects of 48h of water deprivation (WD) or salt loading (SL) in male adult rats on locomotory parameters assessed during 10 min in the open field test. Data were submitted to one-way ANOVA followed by the Tukey post hoc test, except for the distance travelled in the peripheral area, in which the Kruskal-Wallis test was used, followed by Dunn’s post hoc test. Values are mean ± SD. *p<0.05 compared to the control group.
