# Supplementary material for: Water deprivation induces hypoactivity in rats independently of oxytocin receptor signaling at the central amygdala
Source: Front Endocrinol (Lausanne). 2023 Jan 31;14:1062211. doi: 10.3389/fendo.2023.1062211 (PMC9928579; doi:10.3389/fendo.2023.1062211)
Supplement: Supplementary file 4 [file Table_4.docx]

**Supplementary Table 4**. Relative mRNA expression in LHb, BLA and CeA

| **Gene** | **Control** | **48h WD** | **48h SL** | **Statistics** |
| --- | --- | --- | --- | --- |
|  | n=6 | n=6 | n=6 |  |
| **LHb** |  |  |  |  |
| *Slc17a6* | 1.00 ± 0.27 | 1.36 ± 0.61 | 1.13 ± 0.42 | H= 1.684, p=0.457, d.f.=15 |
| *Gad1* | 1.00 ± 0.64 | 1.76 ± 0.72 | 1.42 ± 0.63 | H= 3.029, p=0.231, d.f.=15 |
| *Avpr1a* | 1.00 ± 0.24 | 0.99 ± 0.49 | 0.92 ± 0.16 | H= 0.363, p=0.850, d.f.=15 |
| *Oxtr* | 1.00 ± 0.67 | 1.19 ± 0.56 | 0.94 ± 0.32 | H= 0.947, p=0.640, d.f.=15 |
| **BLA** |  |  |  |  |
| *Slc17a6* | 1.00 ± 0.22 | 1.23 ± 0.44 | 1.36 ± 0.31 | F_(2,15)_= 1.777, p=0.203 |
| *Gad1* | 1.00 ± 0.19 | 0.90 ± 0.17 | 0.95 ± 0.08 | F_(2,15)_= 0.680, p=0.522 |
| *Avpr1a* | 1.00 ± 0.28 | 1.48 ± 0.38 | 1.15 ± 0.33 | F_(2,15)_= 3.276, p=0.066 |
| *Oxtr* | 1.00 ± 0.32 | 1.21 ± 0.31 | 0.96 ± 0.26 | F_(2,15)_= 1.162, p=0.340 |
| **CeA** |  |  |  |  |
| *Slc17a6* | 1.00 ± 0.39 | 0.79 ± 0.30 | 0.95 ± 0.46 | H= 1.450, p=0.512, d.f.=15 |
| *Gad1* | 1..00 ± 0.16 | 1.16 ± 0.24 | 1.04 ± 0.32 | H= 2.140, p=0.360, d.f.=15 |
| *Avpr1a* | 1.00 ± 0.30 | 1.36 ± 0.53 | 1.16 ± 0.31 | H= 2.211, p=0.353, d.f.=15 |
| *Oxtr* | 1.00 ± 0.48 | 2.49 ± 0.81 * | 1.98 ± 0.91 | F_(2,15)_= 5.994, p=0.012 |

Effects of 24h or 48h of Water deprivation (WD) or salt loading (SL) in male adult rats on relative mRNA expression of the Vesicular Glutamate Transporter 2 (*Slc17a6*), the Glutamate Decarboxylase 1 (*Gad1*), the Arginine Vasopressin Receptor 1A (*Avpr1a*), and the Oxytocin Receptor (*Oxtr*) in the lateral habenula (LHb), the basolateral amygdala (BLA), and the central amygdala (CeA). Values are mean ± SD. Data of gene expression in BLA, as well as *Oxtr* mRNA expression in CeA, were analyzed by one-way ANOVA followed by the Tukey post hoc test. The other data were submitted to the Kruskal-Wallis test. *p<0.05 compared to the control group.
