# Supplementary material for: Water deprivation induces hypoactivity in rats independently of oxytocin receptor signaling at the central amygdala
Source: Front Endocrinol (Lausanne). 2023 Jan 31;14:1062211. doi: 10.3389/fendo.2023.1062211 (PMC9928579; doi:10.3389/fendo.2023.1062211)
Supplement: Supplementary file 5 [file Table_5.docx]

**Supplementary Table 5**. Elevated plus maze test

|  | **Control** | | **48h WD** | | **Statistics** | | |
| --- | --- | --- | --- | --- | --- | --- | --- |
|  | Vehicle | Antagonist | Vehicle | Antagonist | Hydration | Injection | Interaction |
|  | n= 8 | n= 10 | n= 9 | n= 9 |  |  |  |
| Closed arm entries (n) | 9.50 ± 2.39 | 8.60 ± 2.12 | 7.00 ± 1.73 | 7.22 ± 1.86 | F_(1,32)_= 8.154, p=0.008 | F_(1,32)_= 0.249, p=0.621 | F_(1,32)_= 0.683, p=0.415 |
| Open arm entries (%) | 44.9 ± 5.9 | 49.6 ± 7.5 | 48.9 ± 10.4 | 47.3 ± 14.9 | F_(1,32)_= 0.073, p=0.789 | F_(1,32)_= 0.198, p=0.659 | F_(1,32)_= 0.837, p=0.367 |
| Time in open arms (%) | 31.7 ± 8.2 | 34.0 ± 10.9 | 26.6 ± 9.0 | 35.9 ± 16.1 | F_(1,32)_= 0.168, p=0.685 | F_(1,32)_= 2.273, p=0.142 | F_(1,32)_= 0.824, p=0.371 |
| Time in central area (%) | 23.0 ± 4.8 | 24.1 ± 6.3 | 25.7 ± 10.22 | 24.0 ± 8.2 | F_(1,32)_= 0.231, p=0.634 | F_(1,32)_= 0.015, p=0.902 | F_(1,32)_= 0.296, p=0.590 |
| Head dipping (n) | 8.13 ± 3.31 | 8.00 ± 3.97 | 8.33 ± 3.35 | 8.89 ± 3.52 | F_(1,32)_= 0.211, p=0.649 | F_(1,32)_= 0.033, p=0.858 | F_(1,32)_= 0.081, p=0.777 |
| Stretch-attend posture (n) | 5.38 ± 2.83 | 6.20 ± 1.87 | 8.89 ± 4.11 | 6.78 ± 4.89 | F_(1,32)_= 1.298, p=0.263 | F_(1,32)_= 0.241, p=0.627 | F_(1,32)_= 1.544, p=0.223 |
| Rearing (n) | 17.5 ± 7.4 | 12.7 ± 6.5 | 15.6 ± 4.3 | 13.3 ± 4.6 | F_(1,32)_= 0.001, p=0.983 | F_(1,32)_= 5.670, p=0.023 | F_(1,32)_= 0.236, p=0.631 |

Effects of oxytocin receptor antagonist microinjection in the central amygdala of 48h water-deprived male adult rats on ethological and exploratory parameters assessed during 5 min in the elevated plus maze test. Values are mean ± SD. Data were submitted to two-way ANOVA. The values of the number of stretch-attend postures and rearing were transformed to ranks before ANOVA.
