# Supplementary material for: Water deprivation induces hypoactivity in rats independently of oxytocin receptor signaling at the central amygdala
Source: Front Endocrinol (Lausanne). 2023 Jan 31;14:1062211. doi: 10.3389/fendo.2023.1062211 (PMC9928579; doi:10.3389/fendo.2023.1062211)
Supplement: Supplementary file 6 [file Table_6.docx]

**Supplementary Table 6**. Water consumption

|  | **Control** | | **48h WD** | | **Statistics** | | |
| --- | --- | --- | --- | --- | --- | --- | --- |
|  | Vehicle | Antagonist | Vehicle | Antagonist | Hydration | Injection | Interaction |
|  | n= 8 | n= 10 | n= 9 | n= 9 |  |  |  |
| Water intake (ml) during 30 min | 1.70 ± 1.08 | 1.24 ± 0.91 | 15.8 ± 2.6 | 15.3 ± 2.46 | F_(1,32)_= 476.4, p<0.001 | F_(1,32)_= 0.468, p=0.499 | F_(1,32)_= 0001, p=0.977 |
| Water intake (ml) during 120 min | 6.70 ± 2.11 | 7.22 ± 1.71 | 30.1 ± 3.9 | 29.6 ± 4.0 | F_(1,32)_= 489.5, p<0.001 | F_(1,32)_= 0.000, p=0.988 | F_(1,32)_= 0.237, p=0.629 |

Effects of oxytocin receptor antagonist microinjection in the central amygdala of 48 h water-deprived male adult rats on water intake during. Values are mean ± SD. Data were submitted to two-way ANOVA.
