# Supplementary figures and images for: Water deprivation induces hypoactivity in rats independently of oxytocin receptor signaling at the central amygdala
Source: Front Endocrinol (Lausanne). 2023 Jan 31;14:1062211. doi: 10.3389/fendo.2023.1062211 (PMC9928579; doi:10.3389/fendo.2023.1062211)

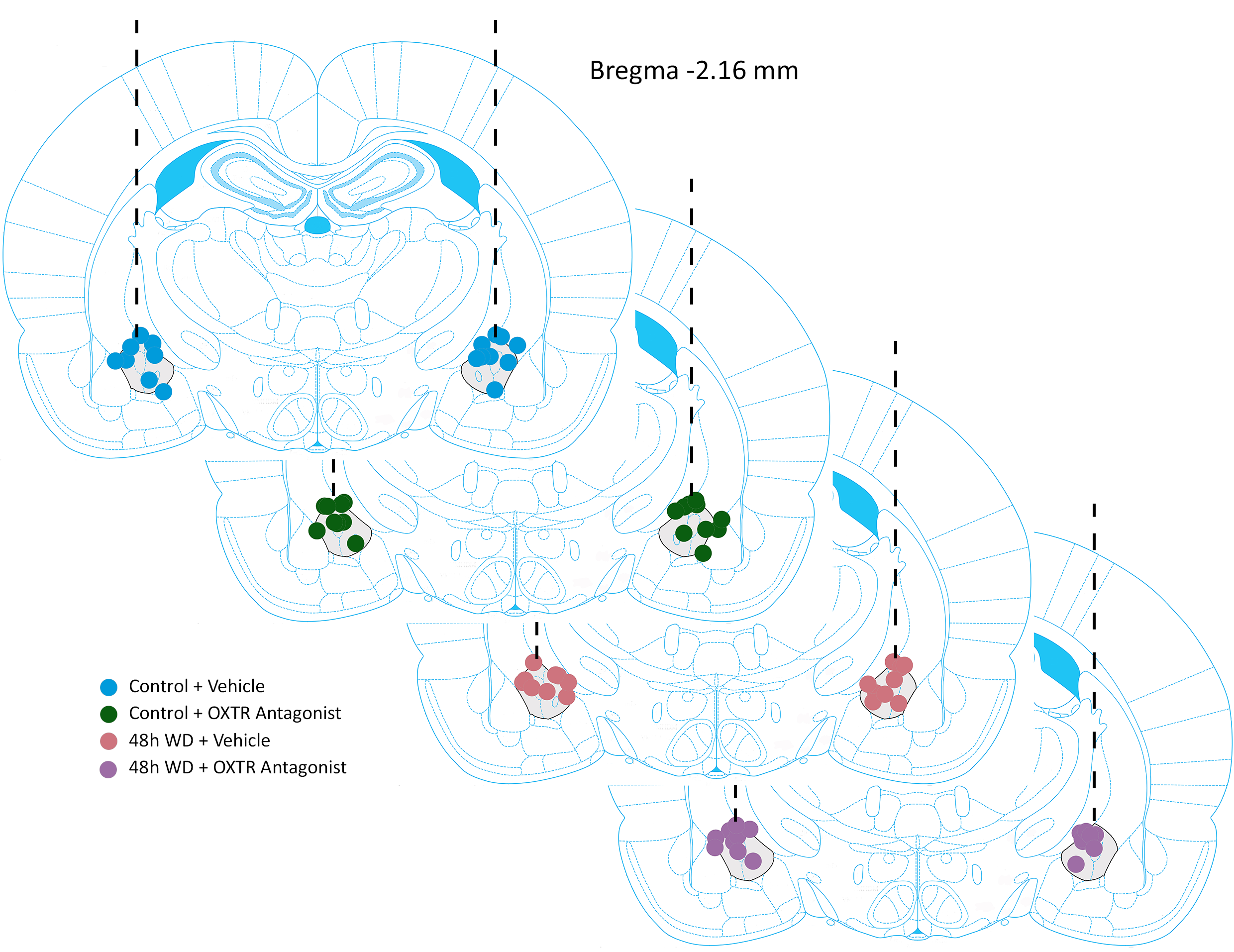

Supplement: Supplementary Figure 1 — Histological location at −2.16 mm posterior to bregma of the cannulas implanted in CeA are shown for control rats administered with vehicle (blue circles) or OXTR antagonist (green circles) and 48-h water-deprived rats administered with vehicle (pink circles) or OXTR antagonist (violet circles). [file Image_1.tif]

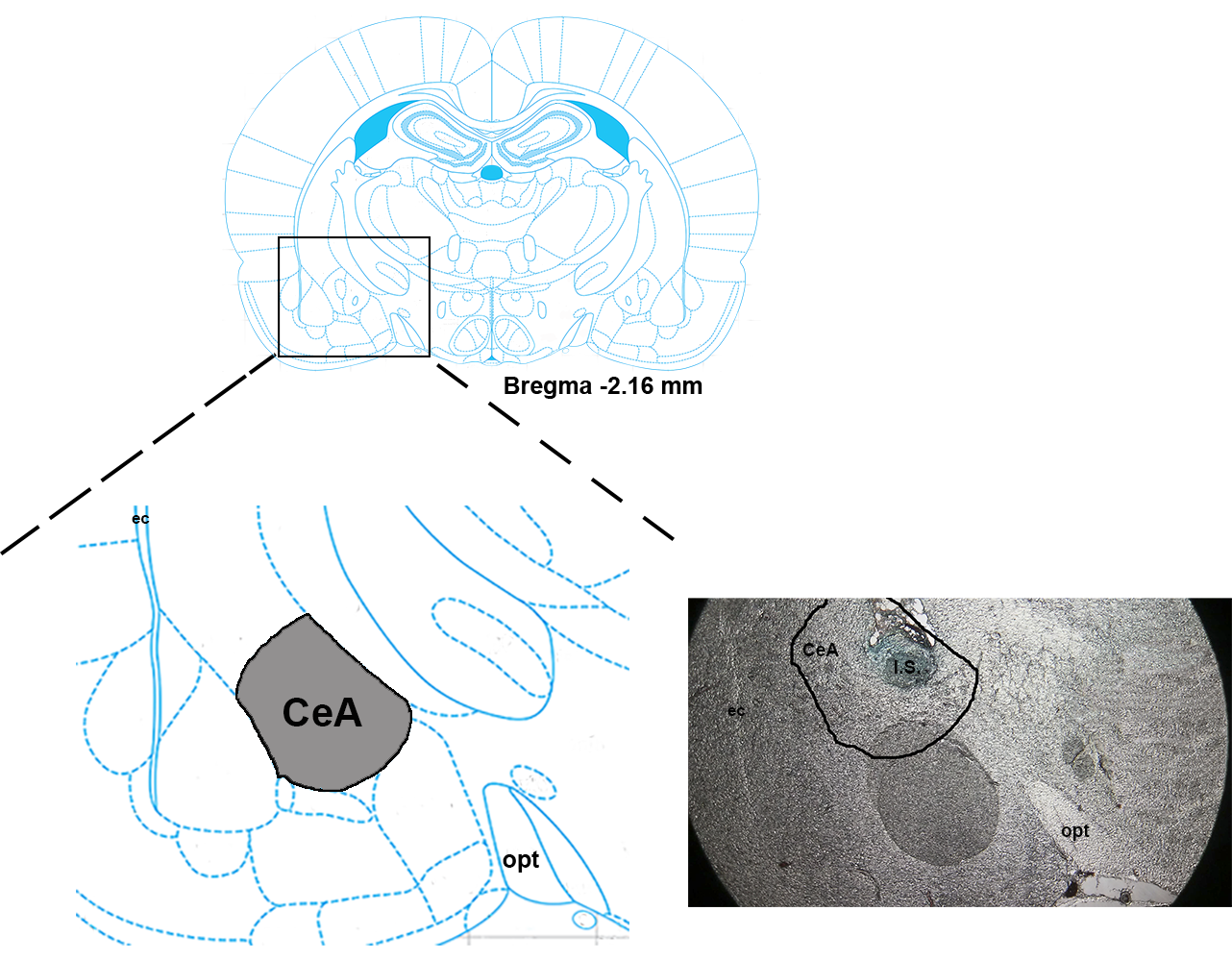

Supplement: Supplementary Figure 2 — Drawing of a coronal section of rat brain at bregma -2.16 mm, and representative microphotograph at 4x showing the microinjection site in the central amygdala. “CeA”, central amygdala; “opt”, optic tract; “ec”, external capsule; and “I.S.”, injection site. [file Image_2.tif]

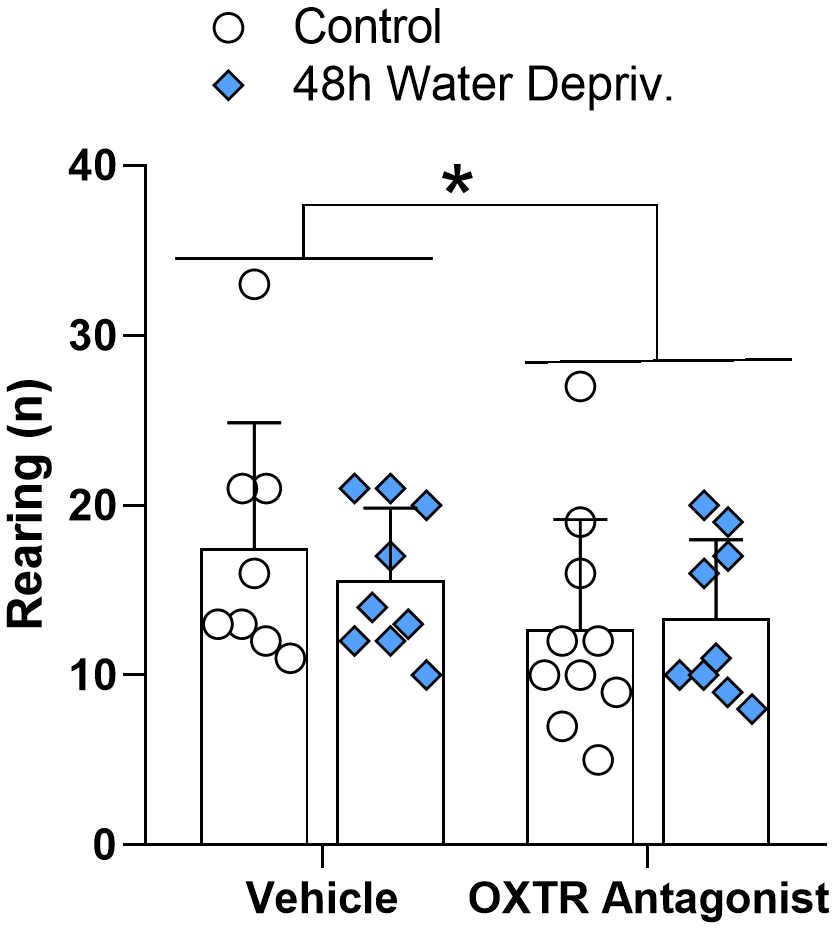

Supplement: Supplementary Figure 3 — Effects of oxytocin receptor antagonist microinjection in the central amygdala of 48-h water-deprived male adult rats on rearing episodes during 5 min of evaluation in the elevated plus maze apparatus. Values are mean ± SD. The number of animals used per group was: Control + vehicle = 8; Control + antagonist = 10; 48 h WD + vehicle = 9; 48 h WD + antagonist = 9. Data were submitted to two-way ANOVA. The values were transformed to ranks before submission to two-way ANOVA. *p<0.05 comparing rats receiving OXTR antagonist vs. rats receiving vehicle. [file Image_3.tif]
